# High-Throughput Biochemical Fingerprinting of *Saccharomyces cerevisiae* by Fourier Transform Infrared Spectroscopy

**DOI:** 10.1371/journal.pone.0118052

**Published:** 2015-02-23

**Authors:** Achim Kohler, Ulrike Böcker, Volha Shapaval, Annabelle Forsmark, Mats Andersson, Jonas Warringer, Harald Martens, Stig W. Omholt, Anders Blomberg

**Affiliations:** 1 CIGENE, Department of Mathematical Sciences and Technology, Norwegian University of Life Sciences, Ås, Norway; 2 Nofima AS, Ås, Norway; 3 Department of Chemistry and Molecular Biology, University of Gothenburg, Gothenburg, Sweden; 4 CIGENE, Department of Animal and Aquacultural Sciences, Norwegian University of Life Sciences, Ås, Norway; 5 Department of Biological and Environmental Sciences, University of Gothenburg, Gothenburg, Sweden; Texas A&M University, UNITED STATES

## Abstract

Single-channel optical density measurements of population growth are the dominant large scale phenotyping methodology for bridging the gene-function gap in yeast. However, a substantial amount of the genetic variation induced by single allele, single gene or double gene knock-out technologies fail to manifest in detectable growth phenotypes under conditions readily testable in the laboratory. Thus, new high-throughput phenotyping technologies capable of providing information about molecular level consequences of genetic variation are sorely needed. Here we report a protocol for high-throughput Fourier transform infrared spectroscopy (FTIR) measuring biochemical fingerprints of yeast strains. It includes high-throughput cultivation for FTIR spectroscopy, FTIR measurements and spectral pre-treatment to increase measurement accuracy. We demonstrate its capacity to distinguish not only yeast genera, species and populations, but also strains that differ only by a single gene, its excellent signal-to-noise ratio and its relative robustness to measurement bias. Finally, we illustrated its applicability by determining the FTIR signatures of all viable *Saccharomyces cerevisiae* single gene knock-outs corresponding to lipid biosynthesis genes. Many of the examined knock-out strains showed distinct, highly reproducible FTIR phenotypes despite having no detectable growth phenotype. These phenotypes were confirmed by conventional lipid analysis and could be linked to specific changes in lipid composition. We conclude that the introduced protocol is robust to noise and bias, possible to apply on a very large scale, and capable of generating biologically meaningful biochemical fingerprints that are strain specific, even when strains lack detectable growth phenotypes. Thus, it has a substantial potential for application in the molecular functionalization of the yeast genome.

## Introduction

A central aim in biology is the understanding of the relation between genetic and phenotypic variation and between gene and function. To address this issue on a larger scale, a variety of analytical methods are in common use. Single-channel optical density measurements of population size have become the preferred methodology for model microorganisms, such as yeast, as net growth in population size is a direct reflection of fitness and therefore of the organisms relation to its environment. However, it is becoming increasingly clear that the population growth parameter space is too insensitive, and too low-dimensional, to reveal much of the inner workings of the cell. Firstly, variations in population net growth in a particular environmental context are often complex phenotypes, making a meaningful decomposition into genetic and molecular components extremely challenging [1]. Secondly, a huge amount of genetic variation fails to leave an imprint on population growth in environments commonly tested. This is problematic because every gene present in yeast has a function that at some point in its recent history has been exposed to purifying selection [2]. This may partially be explained by a failure to recreate natural environments in the laboratory or relaxation of selection through epistatic buffering in the artificial genomes of laboratory strains. However, it is also possible that selection acting on these genes is too subtle to detect with our current methods of quantifying population net growth. It is very possible that removal of such genes, although not leading to a detectable variation in population net growth because of compensatory mechanisms, nevertheless can manifest itself on the molecular and biochemical level. More refined and high dimensional biochemical investigations should be able to reveal such molecular phenotypic signatures which may be used to fingerprint, or even decode, gene function. Transcriptomics, proteomics and metabolomics have all been extensively applied to this aim with varying success rates. Currently, screening of the metabolome is mainly carried out by GC-MS, LC-MS, and NMR spectroscopy. Metabolic fingerprinting [3,4] has been used to classify gene mutants that are silent in terms of growth phenotypes during standard laboratory conditions. Although valuable, metabolic fingerprinting often requires chemical extraction of components before the analysis and protocols are tedious, have a low throughput and are difficult to standardize.

Already in 1998, FTIR (Fourier-Transform Infrared) spectroscopy was proposed as a potentially valuable tool for yeast metabolome analysis [5], but the idea failed to generate substantial interest possibly due to the moderate throughput of existing protocols at this time. The advantage of FTIR spectroscopy is that it can provide a snapshot of the status of whole cells, fluids, and tissues as it reveals a chemical fingerprint which is caused by the sum of all chemical structures within the sample. Ever since the early 1990s vibrational spectroscopy, both Raman and FTIR were used for identification and classification of microorganisms with promising results [6–9]. FTIR provides very high spectral reproducibility and sensitivity: e.g. it has been shown that FTIR spectroscopy can distinguish between different isolates of *Listeria monocytogenes* [10]. Furthermore, IR spectral fingerprints could differentiate between genetically identical bacterial strains growing on different nutritional media [11]. With the current availability of suitable high-throughput instrumentation, IR spectroscopy is gradually emerging as a method of choice for routine analysis in microbial laboratories [12].

As functional characterization of genes/proteins requires screening of large numbers of strains, high-throughput methods with potential for automation are highly needed. There already exist robotized high-throughput phenotyping systems that allow for screening of links between genotypes and organismal phenotypes on a genome-wide scale [13–15]. It is generally accepted that FTIR cannot fully replace a metabolic analysis like GC-MS, LC-MS, or NMR spectroscopy, but it has a high potential for genome-wide screening of thousands of strains which is not realizable with state of the art”wet” chemical methods.

The aim of this study was to develop a high-throughput protocol for large scale biochemical fingerprinting based on FTIR spectroscopy that is robust to both noise and bias. This was addressed by a microcultivation approach with a semi-automated sample preparation procedure to achieve samples of suitable quality for IR measurements on 384-microwell plates. We demonstrate the applicability of this FTIR approach characterizing 76 *Saccharomyces cerevisiae* homozygote diploid gene knock-outs that lack genes in lipid biosynthesis. Many knock-outs of genes annotated as involved in lipid biosynthesis and metabolism lack detectable growth phenotypes under standard laboratory conditions, but may be expected to have subtle changes in overall cellular biochemical composition at levels that could be probed by FTIR. Indeed, we found several of the examined knock-outs to feature characteristic and highly reproducible FTIR phenotypes over the whole spectral range. Conventional analysis of acyl lipid content confirmed deviations in lipid profiles for strains with aberrant FTIR phenotypes suggesting that the spectral data can be causally interpreted with reference to our current understanding of fatty acid metabolism. Our results show the potential of large scale FTIR phenotyping in the detection and interpretation of subtle biochemical effects of genetic variation that is not reflected in population growth aberrations and hints at the potential for large scale FTIR screening of large collections of strains with reverse engineered or natural genetic variation.

## Materials and Methods

### Yeast strains

**Knock-out strains in lipid metabolism.** We used *S*. *cerevisiae* homozygous diploid deletion strains in the BY4743 background with the genotype *MAT* ***a*** */α his3Δ1/his3Δ1 leu2Δ0/leu2Δ0 lys2Δ0/LYS2 MET15/met15Δ0 ura3Δ0/ura3Δ0*, from the EUROSCARF stock center (http://www.uni-frankfurt.de/fb15/mikro/euroscarf/index.html). The analyzed 76 mutants corresponded to knock-outs of genes involved in lipid biosynthesis pathways (see Table 1 for a complete list of analyzed mutant strains).

**Table 1:** Strains used for FTIR phenotyping study.

ORF	Gene	sample #	ORF	Gene	sample #
YHR067W	*HTD2*	1^1^ ^,^ ^2^	YNL045W	*YNL045W*	40
YGR155W	*CYS4*	2	YNR019W	*ARE2*	42
YML075C	*HMG1*	3	YOR011W	*AUS1*	43
YJR150C	*DAN1*	4	YOR049C	*RSB1*	44
WT	*WT BY4743*	5	YOR100C	*CRC1*	45
YJR019C	*TES1*	6	YOR171C	*LCB4*	46
YDR058C	*TGL2*	7	YOR196C	*LIP5*	47^1^
YJL196C	*ELO1*	8	YOR245C	*DGA1*	48^1^ ^,^ ^2^
YKR053C	*YSR3*	9	YOR377W	*ATF1*	49^1^
YCR048W	*ARE1*	10^1^ ^,^ ^2^	YOL002C	*IZH2*	50
YNL130C	*CPT1*	11	YOL011W	*PLB3*	51
YKR067W	*GPT2*	12	YPL147W	*PXA1*	52
YLR450W	*HMG2*	13	YPL057C	*SUR1*	53
YOL101C	*IZH4*	14	YPL006W	*NCR1*	54
YMR313C	*TGL3*	15^1^ ^,^ ^2^	YBL011W	*SCT1*	55
YOR317W	*FAA1*	16	YBL039C	*URA7*	56^1^
YJL145W	*SFH5*	17	YBR030W	*YBR030W*	57
YJL134W	*LCB3*	18^2^	YBR042C	*YBR042C*	58
YJR073C	*OPI3*	19^1^ ^,^ ^2^	YBR159W	*IFA38*	59
YJR103W	*URA8*	20	YBR161W	*CSH1*	60
YKL008C	*LAC1*	21	YBR177C	*EHT1*	61^1^
YKL140W	*TGL1*	22	YBR183W	*YPC1*	62
YKL188C	*PXA2*	23	YDL046W	*NPC2*	63
YLL012W	*YEH1*	24	YDL109C	*YDL109C*	65
YLR023C	*IZH3*	25	YDL142C	*CRD1*	66^1^ ^,^ ^2^
YLR133W	*CKI1*	26	YDR018C	*YDR018C*	67
WT	*WT BY4743*	27	YDR072C	*IPT1*	68
YLR189C	*ATG26*	28	YDR147W	*EKI1*	69^1^
YLR228C	*ECM22*	29	YDR213W	*UPC2*	70
YML059C	*NTE1*	30^2^	WT	*WT BY4743*	71
YML008C	*ERG6*	31^1^ ^,^ ^2^	YDR294C	*DPL1*	72^1^
YMR015C	*ERG5*	32	YDR297W	*SUR2*	73^1^
YMR205C	*PFK2*	33^1^ ^,^ ^2^	YDR492W	*IZH1*	74
YMR207C	*HFA1*	34^1^ ^,^ ^2^	YDR503C	*LPP1*	75^1^
YMR246W	*FAA4*	35	YER044C	*ERG28*	76
YMR272C	*SCS7*	36	YER061C	*CEM1*	77^1^ ^,^ ^2^
YNL323W	*LEM3*	37^1^ ^,^ ^2^	YGL012W	*ERG4*	78^1^ ^,^ ^2^
YNL280C	*ERG24*	38^1^ ^,^ ^2^	YGL126W	*SCS3*	79^1^ ^,^ ^2^
YNL123W	*NMA111*	39	YGL144C	*ROG1*	80

^1^ sample, showed a dominant FTIR phenotype that is different from the wild type after 24 hours of cultivation;

^2^ sample, showed a dominant FTIR phenotype that is different from the wild type after 48 hours of cultivation;

**Natural and industrial yeast strains/species.** A set of 74 strains from four species of the *Saccharomyces sensu stricto clade*, *S*. *mikatae*, *S*. *paradoxus*, *S*. *bayanus*, *S*. *cerevisia*e, as well as from their closest non-*sensu stricto* relative *S*. *kudravzevii*, obtained from Gothenburg University (Gothenburg, Sweden), as well as strains of four species of genus *Candida* (*C*. *tropicalis*, *C*. *intermedia*, *C*. *zeylanoides*, *C*. *inconspicua*), four species of genus *Pichia* (*P*. *anomala*, *P*. *fermentans*, *P*. *stipitis*, *P*. *guilliermodnii*), two species of genus *Hanseniaspora* (*H*. *uvarum* and *H*. *vinea*) and one species of genus *Debaryomyces* (*D*. *hansenii*), obtained from Molecular and General Microbiology Laboratory, UFR Sciences (Reims, France), was used for experimental variability measurements.

### Cultivation of gene knock-out strains for FTIR spectroscopy

76 strains of the *S*. *cerevisiae* homozygote diploid gene knock-outs of the BY4743 series, stored deep-frozen (-80°C) in 20% glycerol, were initially inoculated in 350 μl of SD medium (0.14% yeast nitrogen base without amino acids, 0.5% ammonium sulphate, succinic acid buffered at pH 5.8 and 2% glucose, 20 mg/l histidine, 20 mg/l methionine, 20 mg/l uracil, 20 mg/l lysine, and 100 mg/l leucine) in honeycomb microtiter plates and incubated for ~72 h at 30°C (termed pre-pre-culture). This procedure was repeated once (second incubation ~48 h, termed pre-culture). For experimental runs, pre-cultured strains were inoculated to an optical density OD of 0.03–0.1 in 350 μl of SD medium in honeycomb microtiter plates (as above) and cultivated for either 24 and 48 hours in a Bioscreen C analyzer (Labsystems Oy, Finland). The optical density (OD) was measured using a wide band filter (450–580 nm) and the incubation was set at 30.0°C (±0.1°C) with ten minutes pre-heating time. Plates were subjected to shaking at highest shaking intensity with 60 s of shaking every other minute. OD measurements were taken every 20 minutes. Except where otherwise stated, cell cultures were harvested in the stationary phase (after 24 and 48 h). The cell suspensions were transferred from the 100-well honeycomb plates to 96-well plates (with conical bottom) and the biomass was cleaned from the remaining growth medium by washing 4x with 0.1% NaCl solution in a WellWash AC microtiter plate washer (ThermoScientific, Waltham, MA). After the last washing cycle approximately 50 μl liquid remained in the wells.

### FTIR spectroscopy analysis

After washing, 8 μl of the cell suspension was transferred onto IR-light-transparent Silicon 384-well microtiter plates, which were dried under moderate vacuum (0.9 bar) for 10 to 15 minutes to generate an even thin film suitable for IR measurements. A High Throughput Screening eXTension (HTS-XT) unit coupled to a Tensor 27 spectrometer (both Bruker Optik GmbH, Germany) was used for data acquisition. The spectra were recorded in transmission mode in the spectral region 4000 to 500 cm^-1^ with a resolution of 6 cm^-1^, an aperture of 5.0 mm, taking 64 scans that were subsequently averaged. Prior to each sample measurement, background spectra of the Silicon substrate were collected in order to account for variation in water vapor and CO_2_.

**FTIR phenotypic measurements.** The 76 *S*. *cerevisiae* homozygote diploid gene knock-outs were analyzed by FTIR spectroscopy for two growth times during the stationary phase: 24 hours and 48 hours. The data sets for each time point comprised five replicates of the 76 gene knock-outs and five replicates of three wild type strains (sample 5, 27, 71; see Table 1) of the homozygote diploid wild type strain BY4743, resulting in 390 spectra. For each Bioscreen run five cultivation replicates of 40 strains were prepared for Bioscreen micro-cultivation resulting in 200 cultivations, which were placed on two honeycomb plates with 100 wells each. Thus, the 390 spectra per data set had to be prepared during three Bioscreen micro-cultivations. Measurements were further replicated by a repeated measurement after 3 months to avoid bias from external conditions. This resulted in 4 data sets consisting in 390 spectra each: 24 hours (1), 24 hours (2), 48 hours (1) and 48 hours (2).

**Time measurements.** For studying the effect of cultivation time on the FTIR measurements, 10 strains of the homozygote diploid gene knock-outs of BY4743 including the wild type (Table 1) were selected. The strains were sampled at various stages of fermentative and respiratory metabolism, after 10, 12, 15, 18, 21, 24, 36, 48, 60, and 72 hours of micro-cultivation in the Bioscreen C analyzer. They were then prepared for FTIR spectroscopy as described earlier. Each strain was grown in replicate for each sampling time point, i.e. the data set consisted in 200 spectra altogether.

**Variability measurements.** In order to estimate variability between replicates and repeats, different levels of technical and biological replication were considered. In addition, similarity between strains at different genetic distances, represented by species from rather distant phylogenetic levels, was estimated. For the estimation of the replicate variability, the following variability levels were considered: 1) ***Technical replicate variability*** referring to repeated FTIR measurements using the same cell suspension, which was applied to different sample positions on the silicon well plate; Herein we also consider the same cell suspension in different concentrations expressed in optical density (OD). Different cell concentrations are expected to result in different numbers of cell layers in the films used for FTIR measurements. 2) ***Cultivation replicate variability*** referring to different cultivations obtained in the same Bioscreen experiment and in the same honeycomb plate; 3) ***Honeycomb replicate variability*** referring to replicates obtained from different honeycomb plates but the same Bioscreen run/experiment; 4) ***Experiment variability*** referring to measurements obtained from different experiments (Bioscreen runs). For estimating the similarity between strains at different genetic distances on different following phylogenetic levels were considered 1) population level; 2) species level; and 3) genus level. The similarity was estimated by calculating PCC (Pearson Correlation Coefficient) [16] and was expressed as 1–PCC×10^-4^ for three spectral regions: 3000–2800 cm^-1^, 1800–1500 cm^-1^ and 1200–700 cm^-1^.

For estimating the replicate variability at the different levels, the *S*. *cerevisiae* wt strain from the EUROSCARF stock center was cultivated in SD medium in two honeycomb plates in the Bioscreen C analyzer. The growth and washing procedures were performed as described above. The experiment was performed twice. Variability within different wells of the same honeycomb microtiter plate (Cultivation replicate variability) was estimated by calculating the average PCC (Pearson Correlation Coefficient) for the correlation of each honeycomb-well spectrum with the average spectrum of all spectra of one honeycomb well plate (including 200 spectra). The variability between replicates obtained from two honeycomb microtiter plates of the same experimental run (honeycomb replicate variability) was obtained by calculating the average PCC (Pearson Correlation Coefficient) for the correlation of each honeycomb-well spectrum of both honeycomb-well plates of one experimental run with the respective average spectrum of all spectra of both honeycomb plates (including 400 spectra). The experimental replicate variation was estimated accordingly, considering different experimental runs and calculating the PCC accordingly (including 800 spectra).

In order to estimate variability on different phylogenetic levels, different yeast strain collections were used. The 76 *S*. *cerevisiae* homozygote diploid gene knock-outs from EUROSCARF stock center and *S*. *cerevisiae* from the natural and industrial strains were used to estimate variability within species, between species and between genera.

**Effect of the cell amount on the quality of IR spectra.** *S*. *cerevisiae* was used to study the effect of different cell concentrations on the quality of IR spectra. *S*. *cerevisiae* wt BY4743 was cultivated in 350 μl SD medium in 10 wells of honeycomb plate in the Bioscreen C analyzer. Cultivation was done for 24 hours at 30°C. The washing procedure was performed as described above. After washing was finished, cell suspensions of 10 wells were transferred into one tube. The following serial number of dilutions were performed: 1.5; 1.4; 1.3; 1.2; 1.0; 0.85; 0.8; 0.75; 0.7; 0.65; 0.6; 0.55; 0.5; 0.45; 0.4, and the corresponding cell suspensions were transferred to the FTIR plates.

As optical density ranges we considered three ranges (0.4–0.55, 0.6–0.8, 1.0–1.5). The range 0.6 to 0.8 is used for FTIR spectroscopy. In order to estimate the stability of the protocol also neighboring ranges were considered.

### Lipid analysis

**Cultivation for lipid and parallel FTIR analysis.** Ten of the gene knock-outs with an FTIR distinguishable phenotype and nine of the non-deviating knock-outs together with wild type were selected for lipid analysis. Selected strains were grown in 35 ml medium in Erlenmeyer flasks for 48 h at 30°C with shaking, in order to get enough biomass for GC analysis. After harvest and washing, 8 μl of cell suspension was used for the FTIR analysis and the remaining cell biomass was either used directly or frozen instantly in liquid nitrogen and stored in -40°C for subsequent lipid extraction.

**GC-MS measurements.** The pellets were re-suspended in 1.6 ml ice-cold water and transferred to screw cap glass tubes. Lipids were extracted according to Bligh and Dyer [17], with modifications [18], dried under a stream of nitrogen and re-suspended in chloroform:methanol (2:1). An aliquot was used for analysis total lipid fatty acids and the rest separated by TLC on silica plates impregnated with 0.1% boric acid. The plates were first developed to 2/3 height with chloroform/trietylamine/ethanol/water (30/35/35/6, by vol.) and after drying to the full height with heptane/ethylacetate (50/10, by vol.). Lipids were identified by comparison to chromatographed authentic lipid standard and scraped into screw cap vials. Phospholipid fatty acids were transmethylated and analyzed by capillary gas chromatography as described [19], except dinonadecanoylphosphatidylcholine was used as internal standard.

### Data analysis

**Pre-processing.** All FTIR spectra were pre-processed on the level of the second derivative using a nine point Savitzky-Golay algorithm, in order to enhance the spectral resolution. This was followed by Extended Multiplicative Signal Correction (EMSC) in order to separate physical light-scattering effects as baseline, multiplicative, linear and quadratic wavenumber dependent effects from chemical information in the spectra [20].

**Principal Component Analysis (PCA).** Principal Component Analysis (PCA) was applied for studying phenotypic variation ([21]). PCA aims at decomposing a large number of variables of a data matrix X, into a smaller number of latent variables or principal components (PCs) describing the main variation patterns. Principal components are sorted such that the first PC accounts for the main variation, while the second PC contains the second most variation, and so on. Each principal component (PC) relates to an independent sample variation pattern and an independent variable variation pattern, which can be displayed in score plots and correlation loading plots, respectively. In the score plots usually scores of two components are plotted as scatter plots at a time. In the correlation loading plots, correlation between variables and scores are displayed. When scores and correlation loading plots are studied together for the same pair of components, variable variation patterns can be directly related to sample variation patterns for the respective components [22].

**Partial Least Squares regression.** For calibrating FTIR spectral data for fatty acid measurement by GC analysis, power partial least squares regression (PPLSR) was used [23]. For PPLSR spectra were pre-processed as described above and the spectral regions from 700 cm^-1^ to 1800 cm^-1^ and from 2800 cm^-1^ to 3100 cm^-1^ were selected. For establishing calibration models, the following parameters were calculated: saturated, monounsaturated fatty acids, saturated and monounsaturated phosphatidylcholine (PC) and phosphatidylethanolamine (PE). For establishing calibration models measured fatty acids were standardized by presenting the fatty acids as relative amounts of total fatty acids. This was done in order to avoid too optimistic models due to cross-correlations between single fatty acids and total fatty acids content. In addition ratios of saturated versus monounsaturated fatty acids were used.

All data analysis was done by in-house developed program codes in Matlab 8.0. (The MathWorks Inc., Natick, United States).

### SEM measurements

For scanning electron microscopy the yeast cell samples were prepared in the same way as for FTIR measurements on the Silicon 384-well FTIR microtiter plate and dried at room temperature. The plate was cut into smaller pieces to fit into the specimen chamber of the Zeiss EVO-50-EP (Carl Zeiss SMT Ltd, 511 Coldhams Lane, Cambridge CB1 3JS, UK). The specimen was coated with gold/palladium for 2 x 2 min in a Polaron SC 7640 (Quorum Technologies Ltd., Ringmer, UK) sputter coater to achieve sufficient conductive coating on the sample.

## Results

### High-throughput cultivation and FTIR spectroscopy

**Fig 1:**
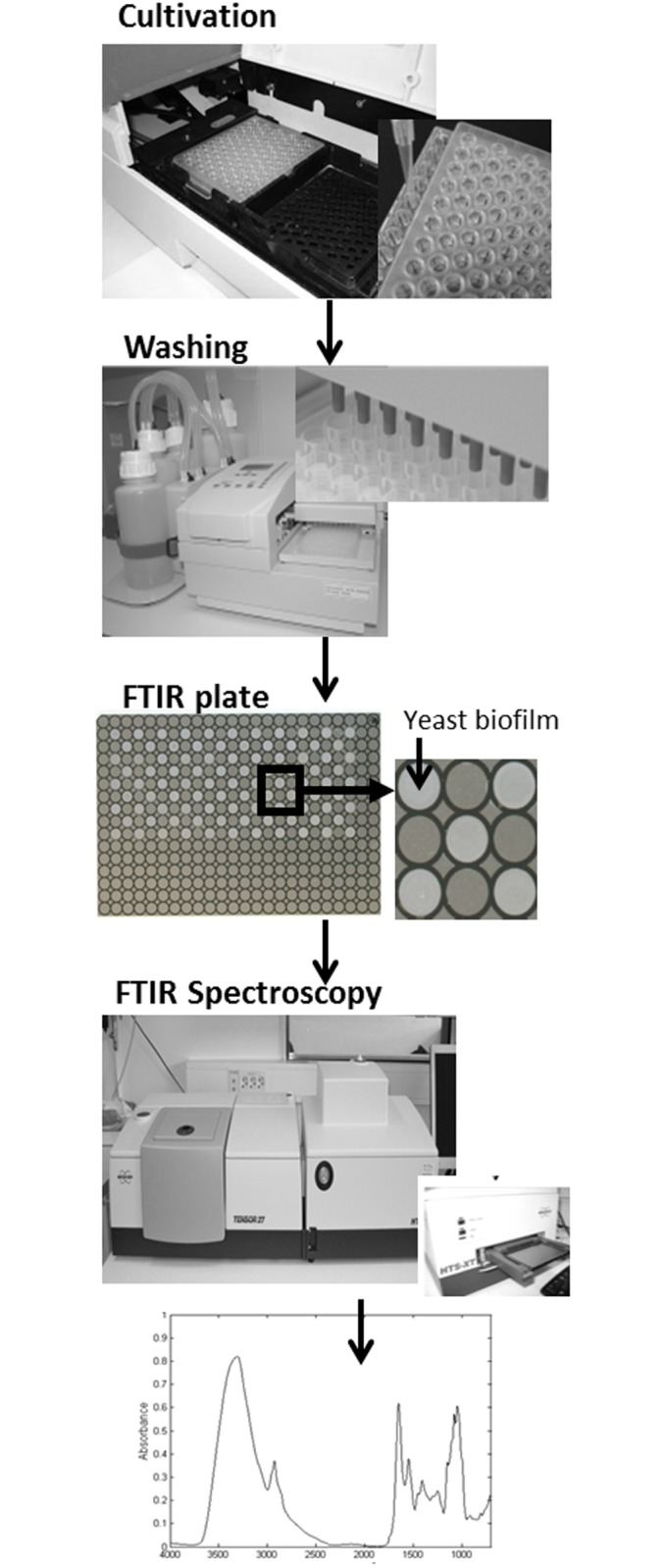
The workflow of sample preparation and high-throughput screening FTIR spectroscopy is shown. In the first step strains were cultivated for 24 or 48 hours in a Bioscreen C microcultivation instrument. Then samples are transferred to a 96-well plate for washing. Washing is performed in WellWash AC microtiter plate washer. At the last step, samples are re-suspended and a film of 8 μl suspension is applied to the FTIR plates. Finally spectra are measured using a Bruker Tensor 27 spectrometer with an eXTension (HTS-XT) unit. The measurement time is approximately 3 hours per plate.

**Fig 2:**
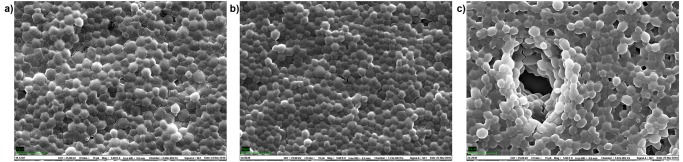
SEM images of films used for FTIR micro-spectroscopy are shown. In (a) and (b) the intact films of strain 19 and the wild type are shown, respectively. In (c) a deficient film formed by a suspension of cells of strain 24 is shown, revealing that the film consists of approximately 8 layers.

A set of 76 *S*. *cerevisiae* homozygote diploid gene knock-outs of genes encoding proteins involved in various aspects of lipid metabolism (Table 1) were selected for examining a developed a high-throughput protocol (Fig. 1) for FTIR spectroscopic phenotyping. These strains are congenic and differ from the wt control only by the absence of a single gene. In Fig. 1, the workflow of the sample preparation and the high-throughput FTIR screening is shown. The basis of the high-throughput phenotyping protocol is the use of well-controlled liquid microcultivation and a growth monitoring system, where yeast strains were cultivated for 24 or 48 hours in a Bioscreen C microcultivation instrument. The microcultivation device allows simultaneous cultivation of up to 200 strains that are grown under the exact same condition. There are a number of technical challenges in the use of FTIR for analysis of yeast, and we here tackle and evaluate them systematically to generate a robust platform for large-scale phenotypic analysis. As further illustrated in Fig. 1, after cultivation, cells were thoroughly washed after growth by a WellWash AC microtiter plate washer. Samples are re-suspended and a film of 8 μl suspension is transferred to IR-transparent plates. Samples are then dried to form a thin film and subsequently measured by using a Bruker Tensor 27 spectrometer with an eXTension (HTS-XT) unit. The measurement time is approximately 3 hours per plate. (see Fig. 1). Different protocols of drying the yeasts into thin cell-films was evaluated using Scanning Electron Microscopy (SEM). In Fig. 2 SEM images of yeast films on FTIR microplates used for FTIR analysis are shown. In Fig. 2a and b two intact films of the knock-out strain 19 (mutant YJR073C, Table 1) and the wt are shown, respectively. In Fig. 2c a defect film formed by the yeast knock-out strain 24 (YLL012W) is shown. The defect area makes it possible to estimate the number of cell layers as approximately 8 (Fig. 2c). This was validated by visually inspecting SEM images of other non-intact films. The complete protocol takes approximately 6 hours for measuring 200 strains. The preparation of 200 samples for FTIR spectroscopy takes approximately three hours, while the fully automatic FTIR measurements per well-plate (with 154 positions covered) took another three hours.

### Time measurements, Growth status

For laying the foundations for a robust and reproducible large scale FTIR phenotyping protocols there is a need for high-throughput cultivation, sample processing and FTIR spectroscopy measurements. A critical issue is the influence of growth status on FTIR readouts as cell populations with different growth properties in a high-throughput set-up will be asynchronous and therefore at the time of harvest in different stages of growth.

**Fig 3:**
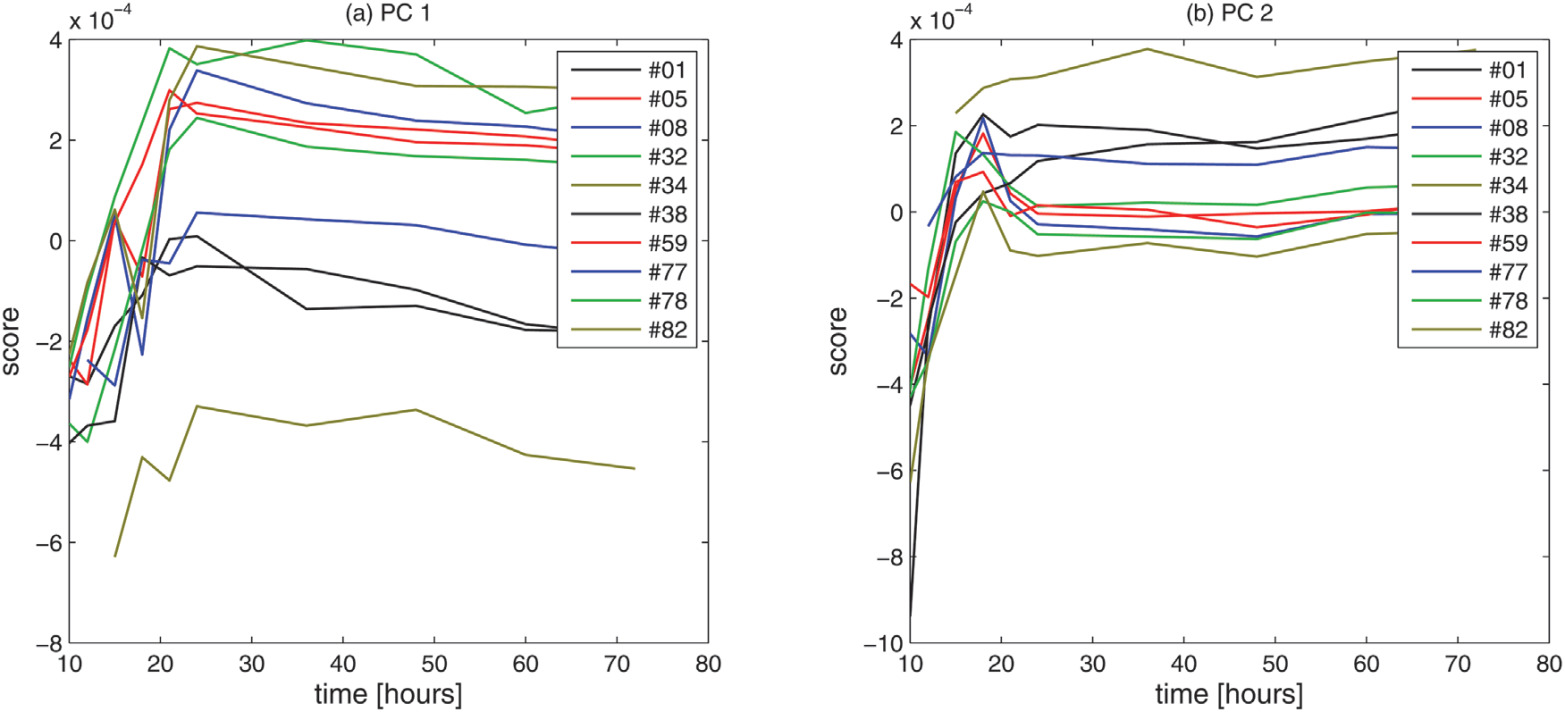
The first and the second scores of the PCA of time measurements of a selection of yeast knock-out strains are shown in (a) and (b), respectively. Samples are measured after 10, 12, 15, 18, 21, 24, 36, 48, 60, and 72 hours. The numbers of the strains are given in the legends.

To investigate the effect of the growth status on the FTIR measurements, 10 strains (including the wt) were sampled at 10 specific growth stages in mid to late exponential phase and early to mid-stationary phase, i.e. after 10, 12, 15, 18, 21, 24, 36, 48, 60, and 72 hours. Spectra were pre-processed and replicate samples were averaged. For sampling at 10 hours, several of the populations provided too few individuals to allow for collecting informative spectra. Thus, the complete dataset consisted of 181 spectra of theoretically 200 possible spectra (2 replicates times 10 strains times 10 time points). To investigate the stability of the FTIR measurements as a function of physiological state (growth stage), principal component analysis (PCA) was used and the first/PC1 and second/PC2 scores were plotted as a function of time. Fig. 3a and b show the scores as a function of time for the first and second component, respectively. Samples are measured after 10, 12, 15, 18, 21, 24, 36, 48, 60, and 72 hours. The numbers of the strains are given in the legends. Overall, scores are unstable during the first 24 hours, but stabilize after 24 hours when virtually all populations have depleted the limiting nutrients and entered the stationary phase. Thus, the robustness of the spectral signature is significantly higher during the stationary phase and that harvesting cells in this phase is to be preferred for FTIR high-throughput phenotyping.

### Reproducibility and Variability of FTIR spectroscopic measurements

Instrumental variability due to light scattering, and sample thickness was minimized using model-based pre-processing EMSC [20], which allows separation of informative signals from spectral artifacts ([11,24,25]).

For the estimation of the remaining variability between technical (the FTIR methodology including washing and drying) and biological (growth) replicates, Pearson’s correlation coefficient (*PCC*) was used. Since the obtained *PCC*-values were very close to one, for comparison and better presentation, results were expressed as 1–*PCC*. Thus, the closer the obtained values were, the lower the variability in the respective data set (details about the calculation of the variability for the different sets investigated are given in the Materials and Methods section). Results are shown in Table 2. Overall, the technical variability was 100 times lower than variability in biological replicates, showing that the robustness of the sample loading, the FTIR measurement and the analysis procedure is excellent. For the technical variability due to differences in the optical density (OD), three different OD ranges were considered (see Table 2). The OD range from 0.6 to 0.8 is the range we use for FTIR spectroscopy, since it refers to an optimal range of the absorbance signal in the amide I, which is usually the strongest absorption band in the FTIR spectra of biological cells. This range shows values for (1–*PCC*) which are close to zero in all spectral regions.

**Table 2:** Variability within technical and biological replicates of *S. cerevisiae* wt. Variability within yeast strains, species and genera.

Type of variability	IR regions		
	3200–2800 cm^-1^	1800–1500 cm^-1^	1200–700 cm^-1^
	(1-*PCC* *)×10^-4^	(1-*PCC* *)×10^-4^	(1-*PCC* *)×10^-4^
Variability within technical replicates**	0.06	0.082	0.64
Variability within IR-spectra for samples with OD 0.4–0.55	0.0–1.0	1.0–5.0	1.0–12.0
Variability within IR-spectra for samples with OD 0.6–0.8	0.0–1.0	0.0–1.0	0.0–1.0
Variability within IR-spectra for samples with OD 1.0–1.5	1.0–6.0	1.0–32.0	1.0–15.0
Variability within biological replicates (Bioscreen wt in run1 on honeycomb plate 1)	4.0	6.3	6.1
Variability within biological replicates (Bioscreen wt in run 1 on honeycomb plate 2)	1.4	1.6	5.4
Variability within biological replicates (Bioscreen wt in run 1 on honeycomb plate 1 and 2)	5.2	8.2	8.3
Variability within biological replicates (Bioscreen wt in run 2 on honeycomb plate 1)	2.2	1.8	6.4
Variability within biological replicates (Bioscreen wt in run 2 on honeycomb plate 2)	7.3	1.7	8.1
Variability within biological replicates (Bioscreen wt in run 2 on honeycomb plate 1and 2)	4.4	2.4	11
Variability within biological replicates (Bioscreen wt in run 1 and run 2)	19	6.9	32
Variability within knock-out *S*. *cerevisiae* strains	87	59	415
Variability within *S*. *cerevisiae* strains from Sanger set	89	40	320
Variability within species*** of genus *Saccharomyces* from Sanger set	78	35	306
Variability within species of genera C*andida*, *Hanseniaspora*,*Pichia*, *Debaryomyces*	105	48	897

All variability tests were done after 24 hours growth.

*Pearson Correlation Coefficient (PCC)

**Technical replicates were obtained from the one sample *S*. *cerevisiae* wt.

***Species included in analysis: *S*. *cerevisiae*, *S*. *paradoxus*, *S*. *bayanus*, *S*. *kudriavzevii*, *S*. *mikatae*, *S*. *castelii* from Sanger set

Moreover, it was observed that different spectral regions have differences in variability. For example, the carbohydrate region 1200–700 cm^-1^ showed the highest variability for technical and for most of the types of biological replicates investigated (see Table 2). The variability between three types of biological replicates (cultivation replicates, honeycomb replicates and experiment replicates, see section 2.3.3) was studied and no difference in variability between the different types of biological replicates was observed (Table 2). Thus, most of the biological variability is due to spatial differences between wells on the same plate, and separating samples in time and instrument does not introduce further noise.

Further variability within different phylogenetic levels (strain, species, genus) was studied. Table 2 demonstrates clearly that variability between strains of the same species is much higher than the variability in biological and technical replicates. The variability within the different phylogenetic levels is about 10–100 times higher than on the replicate level, depending on the spectral region considered. Further we notice that the variability within the set of gene-knock-out *S*. *cerevisiae* strains and the variability in the natural and industrial set of *S*. *cerevisiae* strains was on the same level, despite the much higher genetic variance in the Sanger set (hundreds of thousands of polymorphisms) than in the gene knock-outs set (congenic and in principle only one gene differs; in reality there might be more mutations introduced during the laboratory cultivation, however, there should be less than a hundred variations). The variability within species of the *Saccharomyces* genus from the Sanger set was close to the variability observed on strain level in the Sanger set. At the same time, the variability for the species of *Candida*, *Hansenulaspora*, *Debaryomyces* and *Pichia* genera was significantly higher. The highest variability was observed between genera.

### Strain differentiation

**Fig 4:**
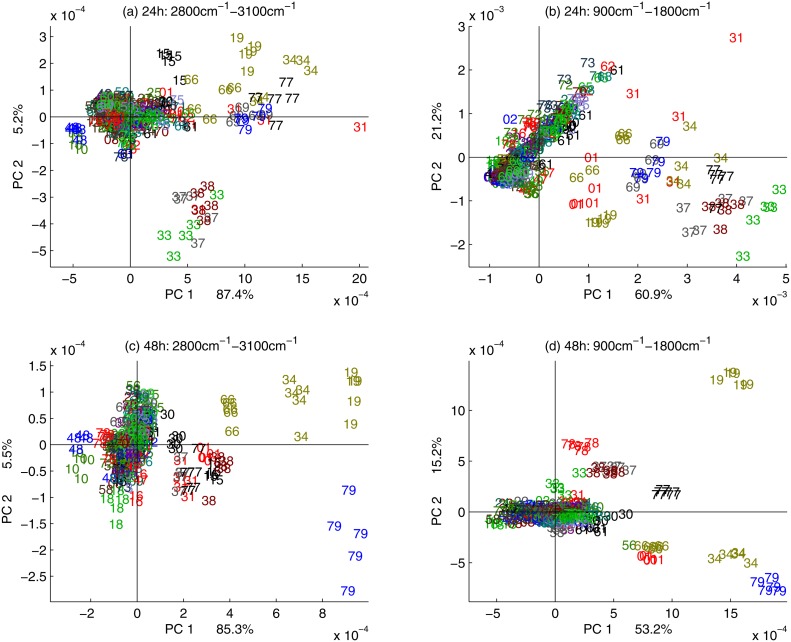
The first and the second scores of the PCA of one experimental run are shown for two harvest time points and two spectral regions. In (a) and (b) the score plots are shown for the spectral region 2800 cm^-1^–3100 cm^-1^ for harvest times 24 hours and 48 hours, respectively. In (c) and (d) the score plots are shown for the spectral region 900–1800 cm^-1^ for harvest times 24 hours and 48 hours, respectively.

To further evaluate the ability of FTIR spectra to distinguish between yeast strains, PCA was performed on each experimental run for 24 hours and 48 hours, separately. For growth on standard medium, the time point 24 hours is at the end of the exponential phase, while the time point 48 hours is in the stationary phase. Each of the experimental runs contained 390 spectra. The score plots for the first and second principal components are shown in Fig. 4a-d, for the two harvest times and two spectral regions, respectively. In Fig. 4a and b the score plots for the spectral region 2800 cm^-1^–3100 cm^-1^ are shown for the harvest times 24 and 48 hours, respectively. In Fig. 4c and d the score plots for the spectral region 900 cm^-1^–1800 cm^-1^ are shown for the harvest times 24 and 48 hours, respectively. Many of the strains show a dominant FTIR phenotype that is different from the wild type both for 24 hours and 48 hours cultivation time. The strains showing a clear phenotype are listed in Table 3. On the PCA score plot it is clearly visible that the FTIR phenotype is highly reproducible: all 5 independent cultivations and FTIR measurements are clustering closely for each deletion strain. Many of the strains exhibiting a distinct phenotype and being different to the wild type after 24 hours also show a distinct phenotype after 48 hours. The 24 hours phenotype and 48 hours phenotype are similar distinctive. Notwithstanding, the level of variation between the independent cultivations of each strain is lower after 48 hours compared to 24 hours growth time. Based on these results and the previous results of time series analysis (Fig. 3), we decided to continue further FTIR spectroscopic analysis with 48 hours cultivation time.

**Table 3:** Strains used for FTIR phenotyping study and GC.

ORF	Gene	sample #
YHR067W	*HTD2*	1^a^ ^,^ ^b^
WT	*WT BY4743*	4
YDR058C	*TGL2*	7
YKR067W	*GPT2*	12
YLR450W	*HMG2*	13
YJR073C	*OPI3*	19 ^a^ ^,^ ^b^
YJR103W	*URA8*	20
YKL140W	*TGL1*	22
YLL012W	*YEH1*	24
YML008C	*ERG6*	31 ^a^ ^,^ ^b^
YMR205C	*PFK2*	33 ^a^ ^,^ ^b^
YMR207C	*HFA1*	34 ^a^ ^,^ ^b^
YMR272C	*SCS7*	36
YNL323W	*LEM3*	37 ^a^ ^,^ ^b^
YNL280C	*ERG24*	38 ^a^ ^,^ ^b^
YOR100C	*CRC1*	45
YBL011W	*SCT1*	55
YDL142C	*CRD1*	66 ^a^ ^,^ ^b^
YER061C	*CEM1*	77 ^a^ ^,^ ^b^
YGL126W	*SCS3*	79 ^a^ ^,^ ^b^
WT	*WT BY4743*	wt

^a^ sample, showed a dominant FTIR phenotype that is different from the wild type after 24 hours of cultivation;

^b^sample, showed a dominant FTIR phenotype that is different from the wild type after 48 hours of cultivation;

**Fig 5:**
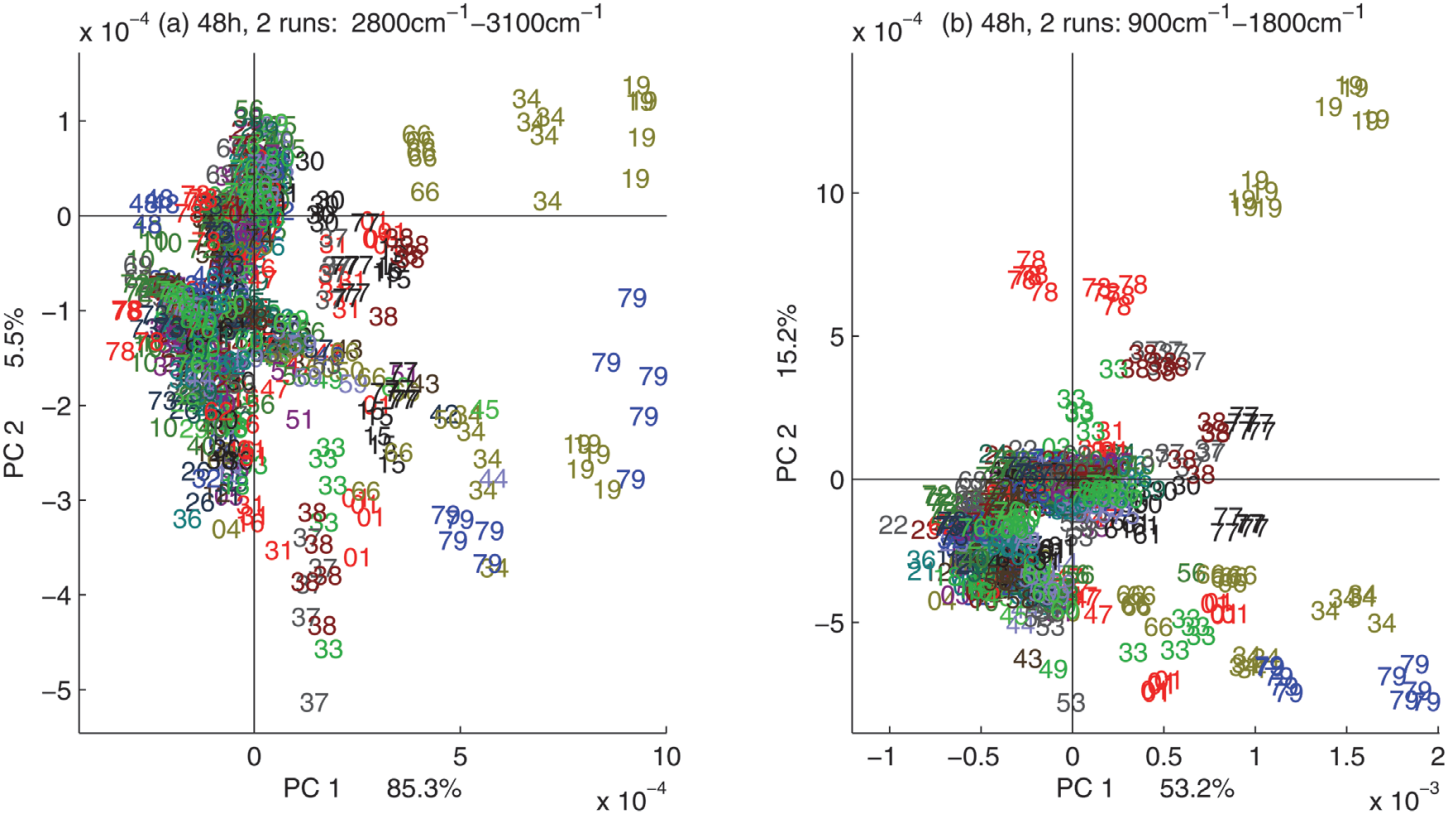
The first and the second scores of both experimental runs are shown for strains harvested after 48 hours for the spectral regions 2800–3100 cm^-1^ and 900–1800 cm^-1^, in (a) and (b), respectively. The principal component model is built on the data collected in run 1for the fatty acid region (2800–3100 cm^-1^) and the region 900–1800 cm^-1^, in (a) and (b) respectively. The data of run two is projected into these models.

When investigating two experimental runs (repeated experiments) in one principal component analysis, a higher degree of variability was observed between the same strains grown in independent experiments. Fig. 5a and b show the score plot using the two experimental runs of strains that were harvested after 48 hours for the regions 3100–2800 cm^-1^ and 1800–900 cm^-1^, respectively. The principal component models were built on the respective spectral region 3100–2800 cm^-1^ (a) and 900–1800 cm^-1^ (b) of the first experimental run and the second experimental runs were projected into these models for each spectral region, respectively. Thus Fig. 5a and b demonstrated to what degree the phenotypes of run 2 are explained by the model built on run 1. Although the variability between replicates originating from different experimental runs is higher than the variability between replicates referring the same run, the same tendencies as in Fig. 5 are clearly observable. Yet, different runs introduce block variation, reflecting bias emerging through separation of samples in time, detectable by the FTIR phenotype.

### Mapping FTIR vs acyl-lipid analysis

**Fig 6:**
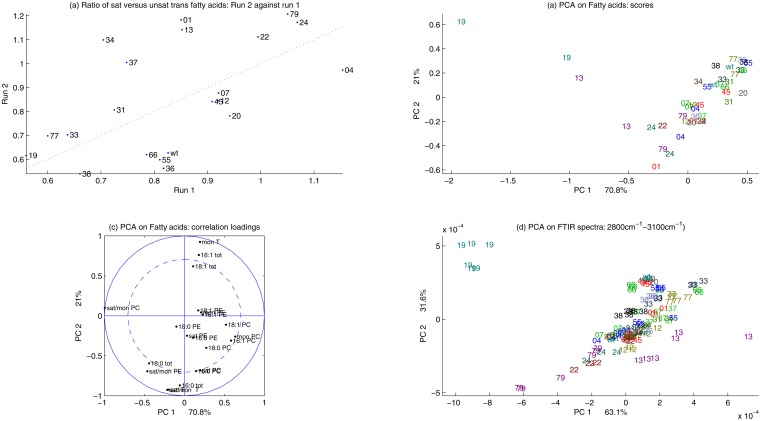
The parallel GC and FTIR results of a selection of samples grown in Erlenmeyer flasks are shown. (a) The experimental run to run variability for the ratio of saturated versus monounsaturated fatty acids is shown. Data are plotted as a scatter plot, where the first experimental run is plotted on the x-axis and the second experimental run is plotted on the y-axis. (b) The first and the second scores of the PCA of the GC analyses are shown. (c) The correlation loading plot corresponding the PCA of the GC analyses is shown (d) The first and the second scores of the PCA of the fatty acid region (2800–3100 cm^-1^) of the FTIR analysis are shown.

To calibrate the FTIR biochemical fingerprint of strains to biochemical information that is directly interpretable from a biological perspective, we analyzed the lipid composition of a subset of deletion strains with distinct FTIR signatures (Tables 4–6). Two independent repeat cultivations of 21 strains were subjected to acyl lipid analysis by GC of fatty acid methyl esters and each of these 42 samples was analyzed in triplicate by FTIR analysis. A total lipid extract was subjected to transmethylation and the total fatty acid content determined by GC. In addition, the glycerolipids were separated by TLC and the major membrane lipid phosphatidylcholine was subjected to transmethylation and analysis of fatty acid methyl esters by GC. The lipid analysis results for the four major fatty acids palmitic (16:1), palmitoleic (16:1), stearic (18:0) and oleic (18:1) are shown in Tables 4 and b, which represent results for both runs separately. It is obvious that there is a considerable variation in the GC measurements from run to run. This variation can be visualized by plotting the results of the two experimental runs against each other. In Fig. 6a the ratio of saturated versus unsaturated trans fatty acids is plotted as a scatter plot with values for run 1 on the abscissa and values for run 2 on the ordinate. The variation between run 1 and run 2 is considerable and even more pronounced than the run to run variation revealed in Fig. 5.

**Table 4:** GC results for total fatty acids.*

	16:0		16:01		18:00		18:1		SUM tot FA	
sample	Run1	Run2	Run1	Run2	Run1	Run2	Run1	Run2	Run1	Run2
1	36.2	40.7	36.5	27.0	9.8	13.5	17.6	18.8	455.7	122.6
4	40.0	38.9	27.8	31.0	13.5	10.5	18.7	19.7	379.9	389.7
7	39.1	37.2	35.8	30.7	8.8	9.5	16.2	22.6	553.7	303.4
12	37.7	38.2	31.4	33.5	10.2	7.6	20.7	20.7	359.1	352.6
13	35.9	39.7	36.2	28.4	10.1	13.6	17.7	18.3	383.6	137.2
19	25.1	27.3	38.0	38.3	10.8	10.9	26.1	23.6	746.9	800.5
20	37.4	35.3	34.6	34.4	11.1	8.5	16.9	21.7	303.8	302.6
22	38.8	40.0	34.9	29.0	11.0	12.6	15.2	18.3	374.5	187.4
24	40.6	42.6	31.4	30.2	11.1	11.4	17.0	15.9	459.6	179.1
31	31.2	31.6	34.4	29.2	10.9	13.0	23.6	26.1	516.3	278.0
33	30.8	31.5	37.1	32.8	8.1	9.7	24.0	26.0	456.0	243.3
34	32.2	41.4	33.2	31.0	9.1	10.9	25.4	16.6	258.2	157.6
36	35.8	28.7	36.8	38.3	9.1	7.3	18.2	25.8	469.2	764.1
37	36.5	41.1	39.3	32.3	6.3	9.0	17.9	17.5	1406.9	469.2
38	29.4	25.6	36.9	38.8	10.3	9.4	23.3	26.2	368.9	425.0
45	38.7	37.3	35.3	37.9	8.9	8.4	17.1	16.4	637.4	407.2
55	36.3	28.4	38.6	35.9	8.5	9.0	16.7	26.6	1104.6	389.3
66	35.7	30.1	41.4	39.3	8.3	8.2	14.6	22.4	1176.8	402.1
77	30.4	32.6	41.2	36.4	7.1	8.6	21.3	22.5	1174.5	688.9
79	40.4	40.5	34.5	26.6	10.8	14.2	14.3	18.7	593.8	259.8
WT	37.5	30.9	37.7	37.6	7.8	7.6	17.1	23.9	600.1	375.7

*-amount of fatty acids is given in nmol;

**Table 5:** GC results for phosphatidylcholine.*

	16:0		16:01		18:00		18:1		SUM PC-FA	
Sample	Run1	Run2	Run1	Run2	Run1	Run2	Run1	Run2	Run1	Run2
1	38.6	37.9	42.2	25.8	6.8	13.8	12.4	22.6	99.4	29.8
4	40.4	39.4	37.8	38.0	8.4	7.5	13.3	15.0	95.7	111.2
7	39.0	39.6	39.8	33.0	9.0	9.4	12.2	18.1	130.2	74.8
12	40.8	39.6	38.7	38.5	6.7	7.5	13.7	14.4	54.2	78.7
13	54.4	45.9	29.0	27.7	10.9	12.8	5.7	13.7	58.4	21.9
19	46.1	51.2	11.9	9.8	20.1	23.4	21.9	15.6	7.0	10.2
20	36.9	25.5	37.2	42.3	9.5	8.1	16.4	24.1	73.0	94.9
22	34.8	44.1	37.4	35.3	9.7	8.7	18.1	11.9	106.2	38.3
24	45.8	37.1	36.0	32.5	8.7	12.9	9.5	17.5	101.1	47.8
31	25.4	19.6	35.9	26.2	13.2	17.8	25.5	36.4	135.7	93.6
33	29.6	29.4	46.3	34.8	6.4	11.3	17.7	24.5	130.7	55.3
34	36.3	33.9	37.1	33.5	10.4	11.7	16.2	20.9	53.6	60.0
36	38.1	28.4	43.8	44.1	7.3	6.7	10.8	20.8	131.9	108.0
37	35.9	39.5	46.9	44.3	5.6	5.4	11.5	10.8	360.3	117.7
38	35.5	19.5	42.2	31.7	8.3	14.8	14.0	33.9	109.2	103.6
45	38.8	31.3	43.5	39.3	7.1	8.1	10.6	21.4	177.2	78.1
55	36.6	25.5	43.0	45.1	7.8	8.1	12.6	21.3	125.6	105.3
66	34.0	25.1	50.0	42.7	5.4	9.5	10.6	22.7	131.3	91.9
77	32.9	28.8	46.6	41.2	5.6	8.6	14.9	21.5	185.7	145.8
79	40.7	42.4	36.7	36.1	9.0	9.8	13.5	11.7	200.4	100.3
WT	36.4	27.5	44.3	36.2	6.5	11.5	12.7	24.8	54.2	77.4

*-amount of fatty acids is given in nmol;

**Table 6:** GC results for phosphatidylethanolamine.*

	16:0		16:01		18:00		18:1		SUM PE-FA	
Sample	Run1	Run2	Run1	Run2	Run1	Run2	Run1	Run2	Run1	Run2
1	28.7	40.4	26.0	32.1	20.8	14.4	24.5	13.1	9.3	9.9
4	21.8	31.3	19.2	29.2	21.1	5.3	37.8	34.1	16.5	45.5
7	19.3	31.9	19.8	33.7	20.6	8.9	40.3	25.5	9.5	31.4
12	34.1	35.9	31.0	30.4	13.4	7.0	21.4	26.7	46.6	23.1
13	18.8	29.0	25.0	27.6	21.5	15.0	34.8	28.4	47.5	19.7
19	25.4	23.5	22.5	34.5	23.5	14.7	28.6	27.3	10.2	30.3
20	35.3	21.4	30.7	30.1	10.9	13.6	23.2	34.9	50.2	33.7
22	31.2	29.5	34.7	30.7	10.5	12.1	23.7	27.7	18.1	15.1
24	41.5	36.5	42.4	22.5	3.9	13.4	12.2	27.6	19.4	8.3
31	21.2	28.3	30.5	23.8	13.0	11.8	35.4	36.2	39.6	9.3
33	17.5	15.5	29.6	25.3	14.2	19.8	38.8	39.4	64.9	21.2
34	18.7	20.5	36.3	32.8	20.2	13.9	24.7	32.8	43.7	48.0
36	38.7	27.8	33.2	40.2	9.9	4.1	18.1	28.0	21.3	37.0
37	32.6	32.7	43.4	38.9	1.9	5.6	22.0	22.7	125.4	55.1
38	25.6	27.0	34.5	29.2	4.1	7.5	35.8	36.3	62.8	15.9
45	33.0	24.5	38.3	37.3	5.1	8.3	23.6	29.9	43.5	68.6
55	29.3	26.6	43.0	46.1	5.3	2.6	22.3	24.7	36.7	47.4
66	30.4	28.7	42.0	39.7	4.1	4.9	23.5	26.7	38.5	43.7
77	25.9	28.5	46.3	43.8	3.1	2.4	24.7	25.3	81.6	65.8
79	31.4	39.6	42.2	36.1	5.4	6.8	21.0	17.4	64.6	23.9
WT	9.3	22.9	26.0	35.0	22.2	9.2	42.5	32.8	5.6	40.7

*-amount of fatty acids is given in nmol;

In Fig. 6 the parallel GC and FTIR results of a selection of samples grown in Erlenmeyer flasks are shown. In Fig. 6a the experimental run to run variability for the ratio of saturated versus monounsaturated fatty acids is shown. Data are plotted as a scatter plot, where the first experimental run is plotted on the x-axis and the second experimental run is plotted on the y-axis. In Fig. 6b and c the score plot and the correlation loading plot of the first and second principal component are shown for the fatty acid analysis. In Fig. 6d the first and the second scores of the PCA of the fatty acid region (2800–3100 cm^-1^) of the FTIR analysis are shown. The PCA sample variation patterns displayed in the score plots for the FTIR results and the fatty acid analyses in Fig. 6b and d shows two main directions. While the variation pattern from the bottom to the upper right is carried by most of the samples, the variation pattern from the right towards the upper left is only borne by very few samples: It is based on the variation caused by the samples 19 (OPI3—Phospholipid methyltransferase (methylene-fatty-acyl-phospholipid synthase), catalyzes the last two steps in phosphatidylcholine biosynthesis) and 13 (HMG2—HMG-CoA reductase; converts HMG-CoA to mevalonate, a rate-limiting step in sterol biosynthesis). In Fig. 6c the cause of the variation pattern is explained in the correlation loading plot. The strongest variation carried only by the samples 19 and 13 is due to the ratio of saturated versus monounsaturated fatty acids in phosphatidylcholine (Table 5). The second and more sustainable variation formed by the rest of the samples is explained by the ratio of saturated versus monounsaturated total fatty acids. In order to compare the variation in the GC data, with the FTIR analyses, we performed PCA on the FTIR spectra of the fatty acid region of FTIR spectra obtained from identical samples as used for the GC measurements. The score plot of the first and second PCA component is shown in Fig. 6c. Surprisingly the PCA of the FTIR fatty acid region in Fig. 6c shows the same variation pattern as the PCA of the fatty acid analysis shown in Fig. 6b. Again the strongest variation pattern is due to difference of the samples 19 and 13, while this time the replicates of the strain 13 lay on the other side of the cloud formed by the rest of the samples. This proves that the fatty acid phenotype as obtained by the GC measurements is captured by the FTIR fatty acid region, i.e. the C-H stretching region from 3100 cm^-1^ to 2800 cm^-1^.

In order to estimate if FTIR spectra can be used to predict measurements of fatty acid composition obtained on identical samples, a PPLSR regression model was established using the FTIR spectra as descriptor variables (**X**) and the fatty acid measurements as independent variables (**Y**). The PPLSR measurements were based on 42 independent GC measurements obtained from independent cultivations. For each sample and GC measurement 3 FTIR replicate spectra were obtained which were averaged before the PPLSR regression. For each GC measurement variable a separate regression model was established. The PPLSR models were validated by full cross-validation where one sample was taken out at the time. The selection of the model size, i.e. the selection of the number of components that were used for the prediction, was very conservative. In most cases only one component was used. The results are shown in Table 7. The prediction results are moderate, which is probably due to the high variability in the GC data. Saturated, monounsaturated (the respective ratio) and the saturated phosphatidylcholine are relatively well explained by the respective prediction models, which mirror the results of the PCA analysis of Fig. 6a, where it was shown that the same fatty acids explain the variation in the GC and FTIR analysis in the first and the second component. For the prediction of the phosphatidylethanolamine by the FTIR spectra no reasonable models could be obtained. The reason for this may be that they are present in very small amounts (see Table 6).

**Table 7:** Prediction results.

PredictionParameters	RMSECV	Mean Y	R2 CV at AOpt	Relative RMSECV %	AOpt
**sat/mon total**	0.2	0.85	0.59	24	1
**sat/mon PC**	0.4	0.92	0.39	43	2
**sat total**	0.06	0.45	0.58	13	1
**mon total**	0.06	0.55	0.58	11	1
**sat PC**	0.04	0.1	0.37	40	1
**mon PC**	0.05	0.13	0.32	38	1

**Fig 7:**
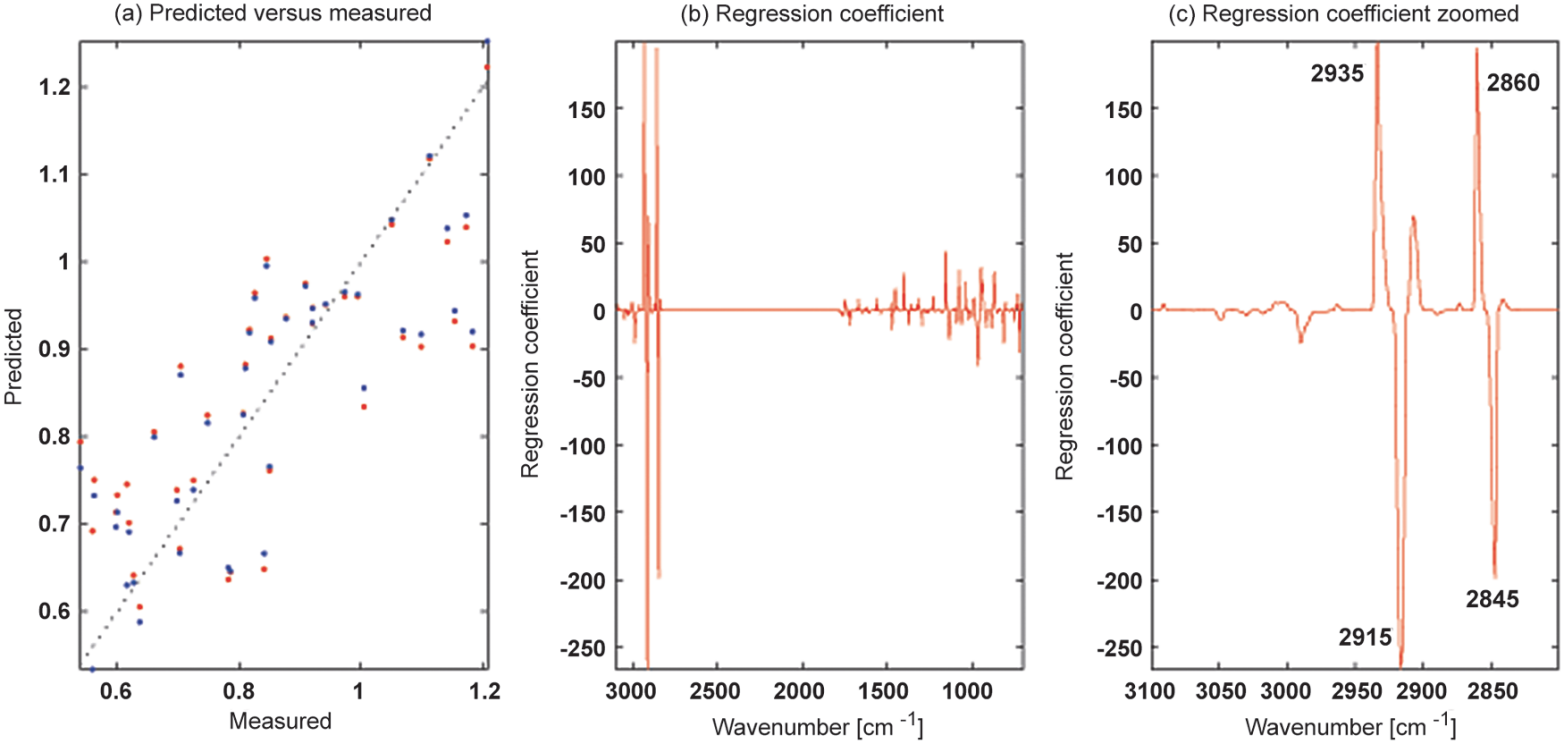
The prediction results for the prediction of GC fatty acids by FTIR data are shown. (a) The predicted ratio of saturated and monounsaturated fatty acids is plotted against the respective GC measurements. For the prediction the spectral regions from 700 cm^-1^ to 1800 cm^-1^ and from 2800 cm^-1^ to 3100 cm^-1^ of the FTIR spectra were used. On the x-axis the measured values and on the y-axis the predicted values are plotted. The calibrated results are shown in blue, while validation results are shown in red. (b) The corresponding regression coefficients are shown. In (c) the fatty acid region from 2800 cm^-1^ to 3100 cm^-1^ is enlarged for the regression coefficient shown in (b).

In Fig. 7a, the predicted ratio of saturated and monounsaturated fatty acids is plotted against the GC measurements. For the prediction the spectral regions from 700 cm^-1^ to 1800 cm^-1^ and from 2800 cm^-1^ to 3100 cm^-1^ of the FTIR spectra were used. On the x-axis the measured values and on the y-axis the predicted values are plotted. The calibrated results are shown in blue, while validation results are shown in red. In order to investigate if the regression model is based on meaningful spectral bands we investigated the regression coefficients. The respective regression coefficients are shown in Fig. 7b and c. In (c) the fatty acid region from 2800 cm^-1^ to 3100 cm^-1^ is enlarged for the regression coefficient shown in (b). The advantage of the PPLSR model compared to ordinary partial least squares regression is that the PPLSR is very selective with respect to spectral bands and thereby improving the interpretability of the model. In Fig. 7b we can clearly see that the regression coefficient has its strongest positive and negative values in the fatty acid region from 3100 cm^-1^ to and 2800 cm^-1^. There are also signatures selected in other spectral regions, which is due to the fact that there are also bands related to fatty acids in the range below 1500 cm^-1^. Fig. 7c shows an enlarged presentation of the fatty acid region from 3100 cm^-1^ to and 2800 cm^-1^. The important bands in this region which are emphasized by the regression coefficient are 2935 cm^-1^ (asymmetric CH_3_ stretch), 2915 cm^-1^ (asymmetric CH_2_ stretch), 2860 cm^-1^ (symmetric CH_3_ stretch) and 2845 cm^-1^ (symmetric CH_2_ stretch). The bands related to CH_3_ bands are positive in regression coefficients while CH_2_ bands are negative. This is meaningful, since second derivative spectra were used in the regression model: In second derivative spectra the bands appear as minima. Thus, a relatively higher share of CH_2_ stretching bands compared to CH_3_ bands in the saturated fatty acids compared to the monounsaturated fatty acids is expressed as positive regression coefficients for CH_3_ bands and negative regression coefficients for CH_2_ bands. This shows that the regression models are meaningful from a chemical point of view.

## Discussion and Conclusion

To investigate the feasibility of large scale FTIR phenotyping to detect subtle biochemical effects arising from known genetic variation with, if at all, marginal consequences on growth, we subjected a selected subset of all viable *S*. *cerevisiae* gene knock-outs to high-resolution FTIR spectroscopy. With few exceptions, these 76 gene knock-outs show no loss of fitness during standard growth conditions, suggesting that the biochemical consequences of gene loss are subtle and inaccessible even to the most precise growth phenotyping technologies. The FTIR signatures of these 76 gene knock-outs were precisely quantified over spectral regions in the FTIR spectra known to predominantly reflect characteristics of distinct biochemical bonds [8]. The spectra were corrected according to an Extended Multiplicative Signal Correction (EMSC) protocol to separate physical light-scattering effects from chemical information in the spectra [20,21], and after this pre-processing spectra are expected to contain predominantly biochemical information. The biochemical information that contributes to the establishment of regression models can be investigated by means of the regression coefficients. It has been shown that the biochemical information contributing to the regression models is meaningful with respect to the fatty acid variables predicted. Yet, it is important to exclude that differences shown in the FTIR phenotype are partially explained by physical variations. Mutant 19 (*OPI3*) which shows a dominant FTIR phenotype for all FTIR measurements (Figs. 4, 5 and 6d) and is obviously different in the saturation of phosphatidylcholine (Fig. 6b and c) was therefore further investigated by SEM analysis and compared to the SEM analysis of the wild type. The SEM analyses were directly performed on the films used for the FTIR measurements (Fig. 2a and b) showing that there are no apparent morphological differences in the cells and the formation of the films used for FTIR analyses. Thus, this gives further confidence that the FTIR signature obtained predominantly contains biochemical information. Since molecular phenotypes of yeast strains strongly depend on growth status [26], the time of harvest for the FTIR analysis is an important issue. In order to harvest yeast strains in a growth status which involves not too strong metabolic changes and thus reduces variability, the stability of the FTIR signal was evaluated as a function of the growth time (Fig. 3). During the first 24 hours of growth strong biochemical changes were detected suggesting against a harvest of the mutants during the first 24 hours. After 24 hours the situation stabilizes, showing that from a variability point of few, strains may be harvested after 24 hours and until 72 hours corresponding to the maximum growth time in the investigated time frame. Nevertheless, the exact time of growth that is finally chosen for the analysis by FTIR or any other method depends on the aim of the study and on the biological question to be answered. But the investigation of the question in focus in this study, i.e. how we should standardize procedures for high-throughput phenotypic analysis of thousands of mutant strains, certainly indicate that 48 hours (stationary phase cells) would be the best.

A key issue for the impact of a large scale effort aimed at the FTIR phenotyping of very large yeast collections is whether the FTIR spectra provide biochemical interpretation that can be transformed into the understanding of gene-function relationships. This, however, is mostly difficult due to the complex nature of FTIR spectra reflecting the summation of all chemical bonds in the cell. Notwithstanding, the causal basis for various spectral bands in the FTIR spectra can be inferred from their co-variation with the detailed measurements of metabolites. As GC signatures for many biochemical compounds are known these can be obtained and compared to FTIR signatures [27,28]. In total, 15 knock-out strains with a distinct phenotype for FTIR measurements as well as 7 strains with no FTIR phenotype plus the wild type BY4743 (2x) were selected for lipid profiling by gas chromatography. Since the growth for GC analysis and parallel FTIR analysis was done in 35 ml medium in Erlenmeyer flasks, it is expected that not exactly the same strains that showed a phenotype in the Bioscreen growth have an FTIR phenotype or a GC phenotype after growth in Erlenmeyer flasks. The growth conditions, including the medium volume and the level of aeration, is expected to influence the phenotype of the mutants [29]. When analyzing the GC and FTIR results of the strains grown in Erlenmeyer flasks, analyses obtained from the same cultivations could be compared. Surprisingly, when comparing the phenotypic variation in the GC analysis with the phenotypic variation in the fatty acid region of the FTIR spectra, identical patterns could be detected (Fig. 6b and d). This strongly suggests that our FTIR experimental setup actually provides relevant and reliable fatty acid information, while being fast and cheap enough to be used for high-throughput screening. It also showed that the levels of specificity in GC and FTIR analysis are comparable, since the two main fatty acid variation patterns in the investigated set of samples could be detected by the FTIR and GC analysis. This main variation patterns were given by the variation in saturated versus unsaturated fatty acids and the variation in saturated versus monounsaturated phosphatidylcholine.

The biological variability in the growth experiments of the yeast knock-out strains is very high. This is revealed both by the GC analysis and the FTIR analysis. In the GC analysis of replicated growth in Erlenmeyer flasks, a strong variability between two different and independent runs could be detected. This was confirmed by the FTIR analysis of the independent runs of the strains grown in Erlenmeyer flasks and by the FTIR analysis of strains grown in the Bioscreen system (Fig. 5). This biological variability seems to be independent from the method used for the chemical and biophysical analysis and in the future it needs to be investigated to what extent it can be further reduced.

The technical variability in the GC analysis is also expected to be high due to the extraction steps involved when isolating fatty acid fractions from yeast strains [30]. Yet, the technical variability of FTIR measurements is very small, as can be seen by the small variability in the replicate measurements of different cultivation grown in the same Bioscreen run (Fig. 4). Thus, FTIR spectroscopy has a very high reproducibility while it is at the same time rather specific.

The disadvantage of FTIR spectroscopy compared to GC analysis and other wet chemical methods is clearly that fatty acid analysis cannot be done independently, i.e. FTIR spectra need to be calibrated against standard analytical methods in order to become biologically interpretable. The consequence is that if there is a high variability in the wet chemical analysis, the calibration model will always suffer from this variability. Yet, the calibration models presented in this paper show that some of the fatty acid variation is clearly captured by both the GC analysis and FTIR analysis and there is a potential of using FTIR spectroscopy to predict some of the most abundant fatty acids in yeast (Table 7).

For improving calibration results further, the biological variability during growth needs to be further reduced and growth conditions need to be standardized. This may be achieved by developing a fully automated growth and harvesting systems involving the different steps of the protocol (Fig. 1). In order to achieve maximum throughput, such a system may be integrated together with the FTIR analysis. We are currently developing a fully automated system for growth and subsequent FTIR analysis of microorganisms in the EU project (http://www.fust.eu.com/), with sufficient throughput for genome-wide phenotyping of gene-knockout strains by FTIR spectroscopy. Using this system enable us to grow and analyze by FTIR spectroscopy 384 samples per day fully automated. Thus, high-throughput FTIR spectroscopy can constitute a substantial technological advancement for functional genomics and can provide for screening of large collections of knock-out strains or other mutant/strain collections.
